# Novel blood pressure and pulse pressure estimation based on pulse transit time and stroke volume approximation

**DOI:** 10.1186/s12938-018-0510-8

**Published:** 2018-06-18

**Authors:** Joonnyong Lee, JangJay Sohn, Jonghyun Park, SeungMan Yang, Saram Lee, Hee Chan Kim

**Affiliations:** 1 Interdisciplinary Program for Bioengineering, Seoul National University Graduate School, Suite 321, Building 8, 101 Daehak-Ro, Jongno-Gu, Seoul, 03080 Republic of Korea; 2 Seoul National University Hospital Biomedical Research Institute, Suite 1203-1, 71 Daehak-ro, Jongno-gu, Seoul, 03082 Republic of Korea; 3 Department of Biomedical Engineering, Seoul National University College of Medicine, Suite 11315, 101 Daehak-ro, Jongno-gu, Seoul, 03080 Republic of Korea; 4 The Institute of Medical and Biological Engineering, Medical Research Center, Seoul National University, Suite 11315, 101 Daehak-ro, Jongno-gu, Seoul, 03080 Republic of Korea

**Keywords:** Blood pressure, Pulse pressure, Stroke volume, Pre-ejection period, Pulse transit time, Ubiquitous healthcare

## Abstract

**Background:**

Non-invasive continuous blood pressure monitors are of great interest to the medical community due to their value in hypertension management. Recently, studies have shown the potential of pulse pressure as a therapeutic target for hypertension, but not enough attention has been given to non-invasive continuous monitoring of pulse pressure. Although accurate pulse pressure estimation can be of direct value to hypertension management and indirectly to the estimation of systolic blood pressure, as it is the sum of pulse pressure and diastolic blood pressure, only a few inadequate methods of pulse pressure estimation have been proposed.

**Methods:**

We present a novel, non-invasive blood pressure and pulse pressure estimation method based on pulse transit time and pre-ejection period. Pre-ejection period and pulse transit time were measured non-invasively using electrocardiogram, seismocardiogram, and photoplethysmogram measured from the torso. The proposed method used the 2-element Windkessel model to model pulse pressure with the ratio of stroke volume, approximated by pre-ejection period, and arterial compliance, estimated by pulse transit time. Diastolic blood pressure was estimated using pulse transit time, and systolic blood pressure was estimated as the sum of the two estimates. The estimation method was verified in 11 subjects in two separate conditions with induced cardiovascular response and the results were compared against a reference measurement and values obtained from a previously proposed method.

**Results:**

The proposed method yielded high agreement with the reference (pulse pressure correlation with reference R ≥ 0.927, diastolic blood pressure correlation with reference R ≥ 0.854, systolic blood pressure correlation with reference R ≥ 0.914) and high estimation accuracy in pulse pressure (mean root-mean-squared error ≤ 3.46 mmHg) and blood pressure (mean root-mean-squared error ≤ 6.31 mmHg for diastolic blood pressure and ≤ 8.41 mmHg for systolic blood pressure) over a wide range of hemodynamic changes.

**Conclusion:**

The proposed pulse pressure estimation method provides accurate estimates in situations with and without significant changes in stroke volume. The proposed method improves upon the currently available systolic blood pressure estimation methods by providing accurate pulse pressure estimates.

## Background

Hypertension, or abnormally high blood pressure (BP), is a major predictor for myocardial infarction, stroke, and other cardiovascular diseases and non-cardiovascular conditions [1–5]. Hypertension can be prevented with careful BP management, but detection and control of hypertension are low prior to diagnosis [6]. Currently, a standard approach for blood pressure measurement uses oscillometric or auscultatory cuffs. However, these devices are rarely used by prehypertensive subjects owing to the lack of availability and convenience. Moreover, the infrequent use of blood pressure cuffs can lead to inaccurate clinical interpretations, as the readings may not be representative of a subject’s true blood pressure values. More frequent BP measurements in patients with hypertension are also recommended for tight BP control [7], but an adequate device has not been developed. Recognition of such needs has attracted much research on developing non-invasive, continuous blood pressure monitoring methods [8, 9].

One of the most promising approaches for non-invasive cuff-less continuous BP monitoring is based on the measurement of pulse transit time (PTT) [10–18]. PTT is the time it takes for a pulse to propagate from a proximal point to a distal point in the arterial tree. PTT has been shown to be physiologically related to BP through pulse wave propagation models [19]. Numerous studies have used this principle to directly estimate systolic BP (SBP), diastolic BP (DBP), and mean BP (MBP), with growing number of studies focusing on increasing the accuracy of such estimation methods by using supplementary strategies. However, attention has not been given to the estimation of pulse pressure (PP), although SBP can be calculated as the sum of DBP with PP and MBP as the sum of DBP and mean PP.

Many methods rooted in the principle of arterial pulse propagation have been proposed for DBP, SBP, and MBP estimation, but proposals for PP estimation are limited. Two recent studies have shown that PP can be estimated using PTT under the pulse wave propagation model with the additional assumption that the volume of blood and the diameter of vessels remain constant in the major arteries that determine PP [20, 21]. However, these assumptions are not valid when significant changes in stroke volume (SV) are involved, and fluctuations in SV occur rather frequently with changes in BP. Therefore, currently available methods based on PTT may be unsuitable for the purpose of PP estimation in many cases.

In this study, we propose a novel method to estimate PP, DBP, and SBP using non-invasive PTT and pre-ejection period (PEP) measured from electrocardiogram (ECG), seismocardiogram (SCG), and photoplethysmogram (PPG). The principle of PP estimation based on SV and arterial compliance (C) is initially explained. PTT and PEP values measured during two hemodynamic interventions in 11 subjects are then used to generate estimations of PP following the proposed principle, and PTT as a sole parameter is used for PP estimation based on the constant volume change PTT-based method. The two estimated PP values are compared to the reference PP measurements, and the errors are analyzed. Subsequently, DBP is modeled using the traditional PTT-based method and added to the PP model to generate estimates of SBP, and the errors in DBP and SBP are analyzed. The performance of the proposed method and the alternative method in the two hemodynamic interventions are discussed with regard to the changes in BP caused by changes in SV and total peripheral resistance (TPR). Finally, potential methods for improving PP estimation and the clinical value of PP as an independent indicator of cardiovascular risk are discussed.

## Methods

### Principles of PP estimation

Considering the difficulty in measuring total arterial compliance C, previous studies proposed the ratio of SV and PP as an indirect measure [22–26] based on the 2-element Windkessel model under the assumption that C is determined by the aorta:

1 $$ \frac{{{SV}}}{{{PP}}} = {C}. $$

Therefore, PP can be estimated if SV and C can be measured using the following equation:

2 $$ {PP} = \frac{{{SV}}}{{C}} . $$

In reality, measuring SV or C is challenging, making it an implausible solution to PP estimation, particularly in terms of non-invasive continuous BP monitoring. However, (2) can be used along with the representation of C using PTT under the pulse wave propagation model along with the assumption that SV can be represented using certain physiological parameters.

Owing to the invasive nature of SV measurement, previous studies used various signals to approximate SV in different settings. Notably, the use of ballistocardiogram (BCG) has been suggested in a few studies [27–29], but the concept has been abandoned because of the lack of accuracy [30]. However, other studies found that PEP is closely related to SV in various situations [31–33]. A study of systolic time intervals reported high correlation between the ratio of PEP and left ventricular ejection time (LVET) with the ratio of SV and end-diastolic volume [34]. More recently, with the development of light-weight high-precision accelerometers, SV estimation based on SCG has gained attraction. A study reported that a linear formula for SV with high accuracy can be achieved using anthropomorphic data, PEP, and SCG features [35], and two studies reported that SV has a linear relationship with PEP [36, 37]. Therefore, in the simplest form, SV may be approximated using a simple relationship given by

3 $$ {SV} \approx {p} \cdot {PEP} + {q}, $$

where $p$ and $q$ represent subject-specific parameters. On the other hand, the time taken for pulse wave to travel, or PTT, along a tube with length $l$ is often modeled as [38].

4 $$ {PTT} = {l} \cdot \sqrt {\frac{{\rho}}{{A}} \cdot {C}_{{{tube}}} ,} $$

where *ρ* represents fluid density, *A* represents the cross-sectional area of the tube, and *C*_*tube*_ represents the compliance of the tube. Therefore, the compliance of the tube can be modeled as follows:

5 $$ {C}_{{{tube}}} = \frac{{A}}{{{\rho}\cdot {l}^{2} }} \cdot \left( {{PTT}} \right)^{2} . $$

As the 2-element Windkessel model assumes the circulatory system as a single tube with its characteristics dominated by the large arteries, we can assume that the systemic compliance in (2) is approximately equal to the compliance of the tube in (5), and we obtain the following:

$$ {PP} \approx \frac{{{p} \cdot {PEP} + {q}}}{{\frac{{A}}{{{\rho}\cdot {l}^{2} }} \cdot \left( {{PTT}} \right)^{2} }} $$

6 $$ {PP} \approx{\beta}_{1} \cdot \frac{{{PEP}}}{{{PTT}^{2} }} +{\beta}_{2} \cdot \frac{1}{{{PTT}^{2} }} +{\beta}_{0} . $$

Hence, PP can be represented as a linear combination of PEP and PTT with subject-specific parameters *β*_0_, *β*_1_, and *β*_2_.

### Principles of DBP and SBP estimation

A popular model for BP is derived from (4) with C as function of BP, and is given as follows:

7 $$ {BP} = \frac{{{\alpha}_{1} }}{{{PTT}}} +{\alpha}_{0} . $$

The above model is often used for both DBP and SBP; however, due to the wave reflection interference, it is assumed to better correspond with DBP [19]. Therefore, DBP can be estimated using (7), PP can be estimated using (6), and SBP can be estimated as the sum of the two estimates:

8 $$ {SBP} = {DBP} + {PP} $$

### Hardware

**Fig. 1:**
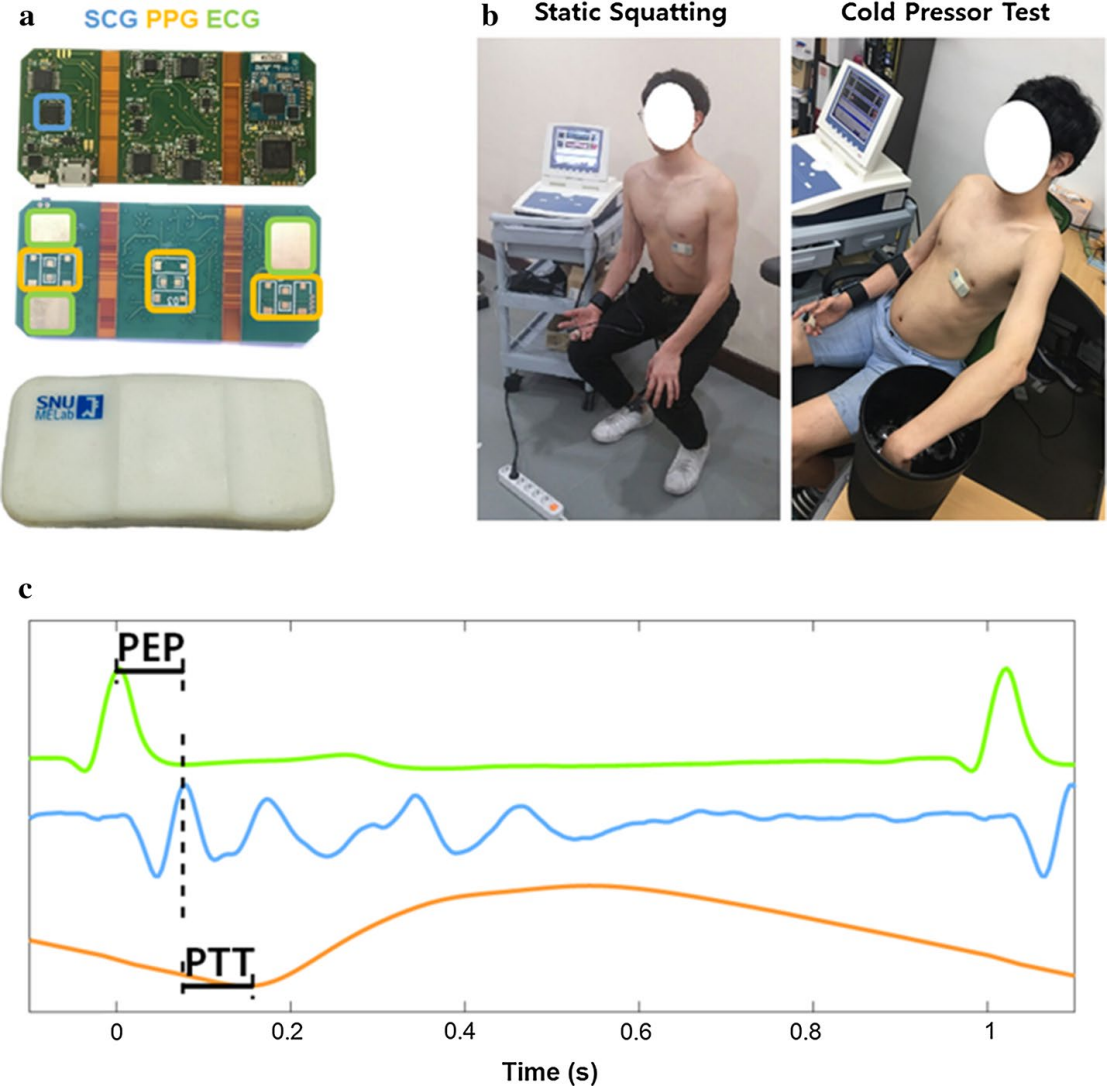
**a** Image of the custom-developed hardware for bio-signal acquisition. The ECG electrodes are boxed in green; SCG accelerometer, light-blue; and PPG LED-PD pairs, orange. **b** Image of a subject performing the two hemodynamic interventions; static squatting on the left and cold pressor test on the right. **c** A sample data of ECG (green), SCG (light-blue), and PPG (orange) with PEP labeled in between ECG R-peak and SCG AO-peak and PTT labeled between SCG AO-peak and PPG waveform minimum. *ECG* electrocardiogram, *PPG* photoplethysmogram, *SCG* seismocardiogram, *LED* light-emitting diode, *PD* photodiode, *AO* aortic opening, *PEP* pre-ejection period, *PTT* pulse transit time

Custom hardware was developed to acquire ECG, SCG, and PPG from the torso (Fig. 1a). The ECG channel was composed of three electrodes, one ground and two forming a single-lead, and an analog-front-end (AFE) for filtering and amplification. Hydrogel patches were placed over the ECG electrodes to improve the signal-to-noise ratio (SNR). The signal from the lead electrodes is passed through an instrumental amplifier (INA333; Texas Instruments, Dallas, TX, USA) for amplification by a factor of 200. The signal is then passed through a passive high-pass filter with a cutoff frequency of 0.3 Hz, followed by a second-order Butterworth low-pass filter with a cutoff frequency of 40 Hz.

The PPG channel was composed of three pairs of light-emitting diodes (LED) and photodiodes (PD) connected to a current-to-voltage converter and an AFE. The PPG signal was passed through a passive high-pass filter with a cutoff frequency of 0.3 Hz, followed by a second-order Butterworth low-pass filter with a cutoff frequency of 20 Hz.

The SCG channel was composed of an analog accelerometer (ADXL327; Analog Devices, Norwood, MA, USA) with a sensitivity of 420 mV/g, followed by a passive first-order low-pass filter with a cutoff frequency of 22 Hz. Although there were three outputs for the accelerometer, only the anteroposterior axis signal was acquired for SCG analysis.

The three analog signals were digitized using a 16-bit sigma-delta analog-to-digital converter (ADC) (STM32F373; STMicroelectronics, Geneva, Switzerland). The data were acquired at 250 Hz and sent to a computer for data analysis using a Bluetooth 4.0 module (BoT-CLE110; Chipsen, Seoul, South Korea).

### Subjects

Eleven healthy young males (27.1 ± 1.5 years old) without any history of cardiovascular conditions were recruited for this study. All subjects provided informed consent. The study was approved by the Institutional Review Board of Seoul National University Hospital.

### Experiment

#### Setup

The custom hardware device was placed over the skin of the sixth left costal cartilage. The device was attached to the skin using double-sided medical tape (1522, 3 M, Maplewood, MN, US) and fixed in place using a medical tape (Hypafix; BSN Medical, Hamburg, Germany). A continuous BP monitor based on the volume clamp method (Finometer; Finapres Medical Systems, Enschede, Netherlands) was used as a reference. The cuff from the reference device was worn around the right index finger, and it was calibrated with each new measurement using the auscultation method. The data from the reference device were collected using a commercial ADC (NI USB-6009; National Instruments, Austin, TX, USA) at 250 Hz and then synchronized with the data from the developed device in a custom data acquisition program (LabView 2013; National Instruments, Austin, TX, USA).

#### Static exercise

Static squatting exercise was performed to induce hemodynamic change based on increased cardiac output (CO) and increased TPR [39]. Subjects stood still without movement for a 30-s rest-state recording. The subjects were then asked to squat against a wall with the knees flexed at 90 degrees with the arms placed comfortably against the torso until SBP was raised by 40 mmHg or until 2 min passed (Fig. 1b). Subsequently, the subjects stood up for a recovery phase until SBP returned to pre-exercise level. The data from standing up and squatting down were omitted due to large movement artefacts.

##### Cold pressor test

Cold pressor test was performed to induce hemodynamic change based on increased TPR [40, 41]. The subjects sat on a chair with arms placed on the sides for 30-s rest-state recording. The subjects then immersed their left hand in ice water at 4 °C for 2 min (Fig. 1b). Subsequently, the subjects removed their hand for a recovery phase until SBP returned to the baseline level.

### Data analysis

#### Pre-processing

All data processing was performed using MATLAB (MATLAB 2016b; MathWorks, Natick, MA, USA). ECG, SCG, PPG, and BP waveforms in 10 cardiac cycles were ensemble averaged in a moving window manner to improve SNR and to accentuate waveform features. PEP was extracted between ECG R-peak and SCG AO-peak, and PTT was extracted between SCG AO-peak and PPG waveform minimum (Fig. 1c). Although PEP is traditionally defined by the onset of the ECG Q-wave, R-peak was used for accurate extraction in this study. From the BP waveform, SBP was extracted as the maximum, and DBP was extracted as the minimum. PP was calculated as the difference between SBP and DBP. All parameters were then smoothed using a 20-beat smoothing window. On average, about 110 cardiac cycles of PEP and PTT values were extracted per subject in each intervention.

#### Linear models

To create a linear model of PP using (6), PEP/PTT^2^ and 1/PTT^2^ were used as input parameters for PP. To test the proposed method against the methods proposed by Ding et al. [20] and Tang et al. [21], a linear model of PP using 1/PTT^2^ as the sole input parameter was also generated, as described in (9).

9 $$ {PP} \approx {m}_{1} \cdot \frac{1}{{{PTT}^{2} }} + {m}_{0} . $$

For SBP estimation, PTT was used as the sole parameter for DBP modeling as described in (7), and the PP estimate was added to the DBP estimate as described in (8).

For all linear models, Least Squares method was used to derive the corresponding coefficients.

#### Performance of the proposed PP model

PP estimates using the proposed method and the PTT-based method were compared with the reference Finometer PP in terms of correlation and agreement. Pearson’s correlation coefficient between Finometer PP and PP estimates were calculated. Bland–Altman plots were generated for both methods indicating the mean difference and the limits of agreement.

The data from each subject were separated into 5 parts randomly to use 4 parts for generating the models and 1 part for validating the models in a fivefold cross validation manner. Root-mean-square error (RMSE) was calculated for the PP, DBP, and SBP models in all subjects using the validation data. The proposed PP estimation method using PEP and PTT was compared against the method using PTT as the sole input in terms of mean RMSE values across all subjects. The normality of the mean RMSE values from the proposed PP model and the PTT-based PP model were verified using the Kolmogorov–Smirnov Normality Test, and the difference in the mean RMSE values was analyzed using Student’s *t*-test. A *p* value < 0.05 was considered statistically significant.

## Results

### PP linear models

**Fig. 2:**
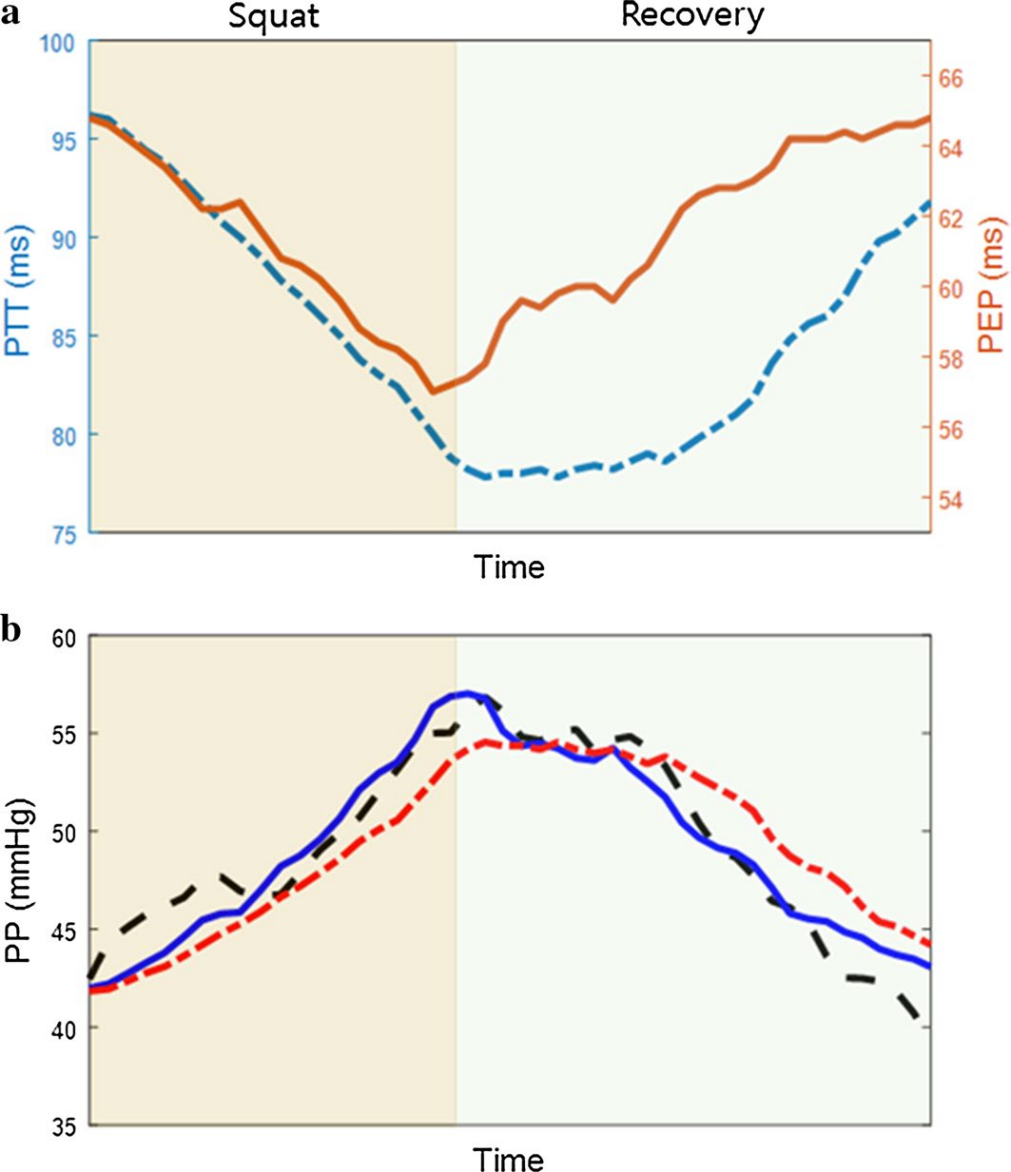
**a** PTT and PEP measured from one subject during (squat) and after (recovery) static squatting exercise. PTT is indicated in a blue dash-dot line, and PEP is indicated in an orange solid line. **b** Reference PP from Finometer (black dashed line), PTT-based estimated PP (red dash-dot line), and estimated PP using the proposed method (blue solid line). *PP* pulse pressure, *PTT* pulse transit time, *PEP* pre-ejection period

**Fig. 3:**
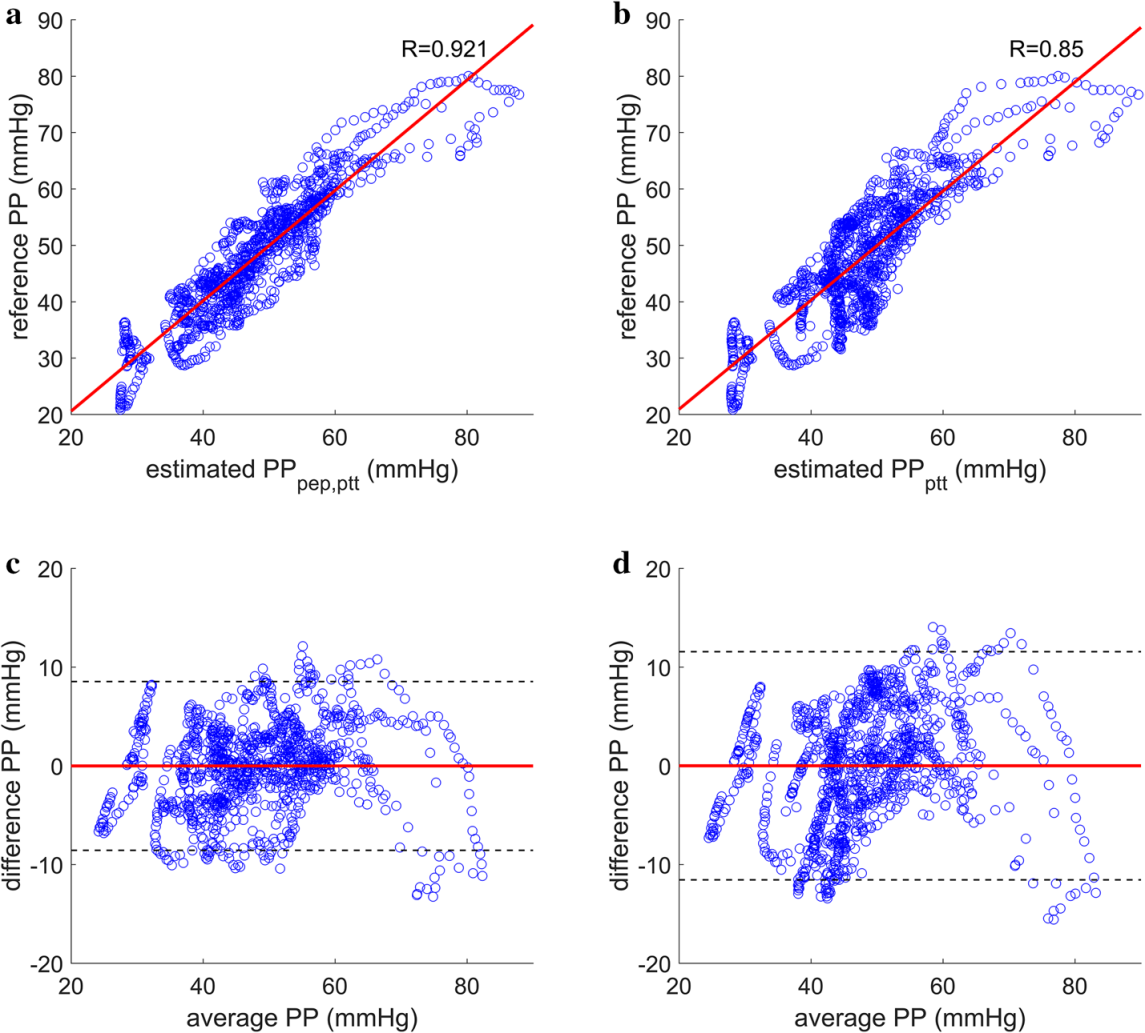
Correlation plots and Bland–Altman plots for PP estimation during static squatting exercise. **a** Reference PP plotted against estimated PP using the proposed method (R = 0.927). **b** Reference PP plotted against estimated PP using the PTT-based method (R = 0.851). **c** Bland–Altman plot of the estimated PP using the proposed method with mean difference marked in solid red line and ± 1.96 SD marked in dashed black lines. **d** Bland–Altman plot of the estimated PP using the PTT-based method with mean difference marked in solid red line and ± 1.96 SD marked in dashed black lines. *PP* pulse pressure, *PEP* pre-ejection period, *PTT* pulse transit time, *SD* standard deviation

**Fig. 4:**
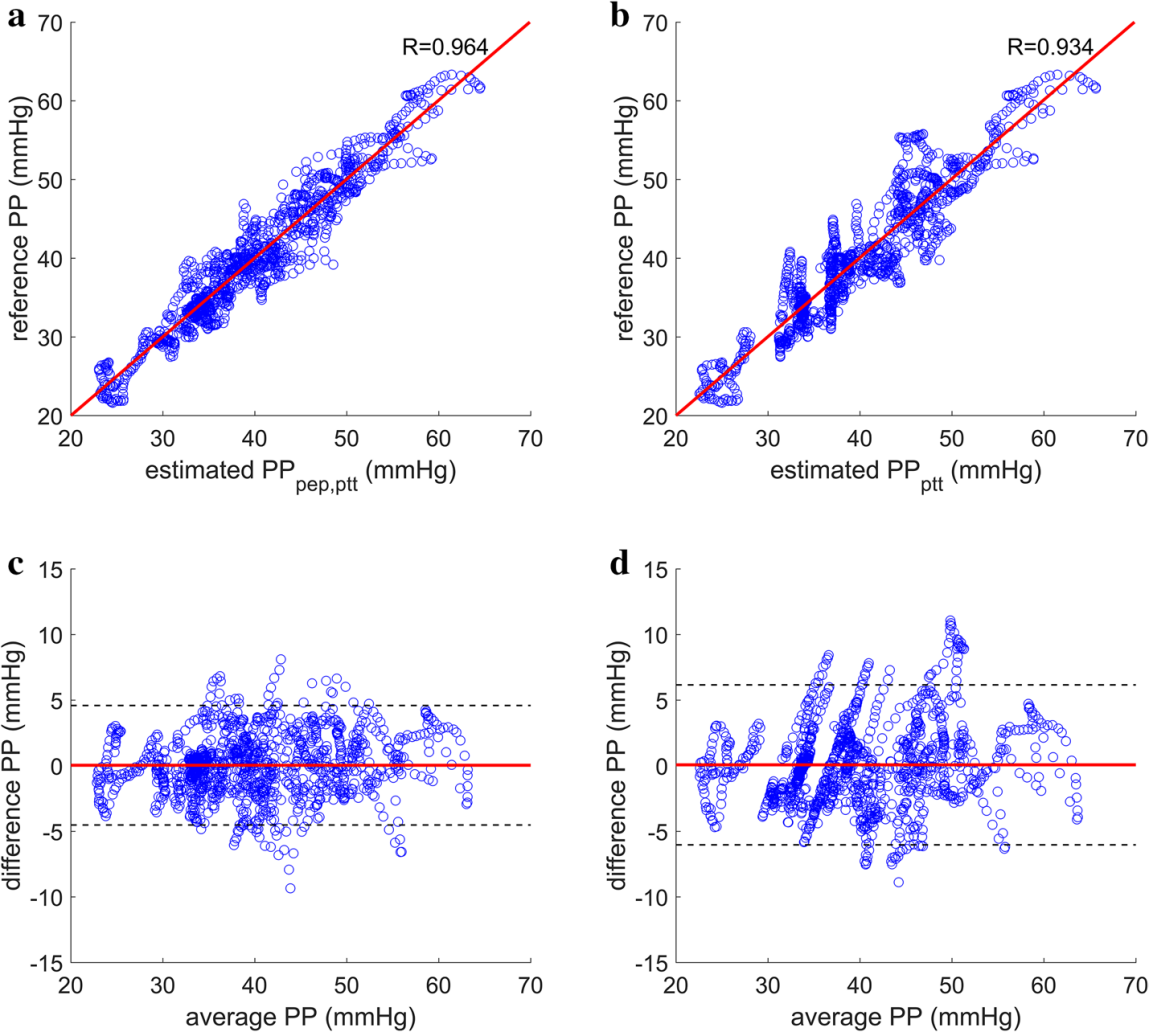
Correlation plots and Bland–Altman plots for PP estimation during cold pressor test. **a** Reference PP plotted against estimated PP using the proposed method (R = 0.964). **b** Reference PP plotted against estimated PP using the PTT-based method (R = 0.934). **c** Bland–Altman plot of the estimated PP using the proposed method with mean difference marked in solid red line and ± 1.96 SD marked in dashed black lines. **d** Bland–Altman plot of the estimated PP using the PTT-based method with mean difference marked in solid red line and ± 1.96 SD marked in dashed black lines. *PP* pulse pressure, *PEP* pre-ejection period, *PTT* pulse transit time, *SD* standard deviation

An example of changes in PEP and PTT and the resulting PP models are shown in Fig. 2. Correlation plots and Bland–Altman plots for the proposed method and the method based on PTT are shown in Figs. 3 and 4. The proposed method for PP estimation yielded Pearson’s correlation coefficients of 0.927 and 0.964 for static exercise and cold pressor test, respectively, whereas the PTT-based method for PP estimation yielded Pearson’s correlation coefficients of 0.851 and 0.934 for static exercise and cold pressor test, respectively. The mean differences between the reference PP and the estimates from both methods were extremely close to zero (i.e., < 0.0001 mmHg), and the limits of agreement, 1.96 standard deviation (SD), were smaller for the proposed method in both experiments compared with the PTT-based method.

The proposed method for PP estimation yielded mean RMSE values of 3.46 mmHg for static exercise and 2.19 mmHg during cold pressor test across all subjects. On the other hand, the method proposed in [20, 21] yielded mean RMSE values of 4.98 and 2.87 mmHg across all subjects during static exercise and cold pressor test, respectively. The difference in mean RMSE values yielded statistically significant improvement in the proposed method in both hemodynamic interventions at *p *< 0.05 (Table 1).

**Table 1:** Accuracy of estimated PP based on PTT, and PP estimated using the proposed method

Subject	Static exercise RMSE (mmHg)		Cold pressor test RMSE (mmHg)	
	Proposed method	PTT-based method	Proposed method	PTT-based method
1	3.87	6.99	1.71	2.08
2	2.22	2.16	3.14	6.43
3	4.20	6.38	2.63	3.12
4	5.89	7.22	2.23	2.31
5	1.49	3.08	0.66	0.85
6	6.04	7.62	2.95	2.99
7	1.53	5.96	1.95	3.26
8	2.07	3.22	1.61	1.60
9	6.06	6.03	3.14	3.87
10	4.49	4.55	1.87	1.96
11	1.58	2.51	2.76	3.35
Mean	3.59^a^ ± 1.89	5.07 ± 2.02	2.24^b^ ± 0.77	2.89 ± 1.47

^a^Statistically significant difference at *p *< 0.01 compared with the PTT-based method

^b^Statistically significant difference at *p *< 0.05 compared with the PTT-based method

### DBP and SBP estimation results

**Fig. 5:**
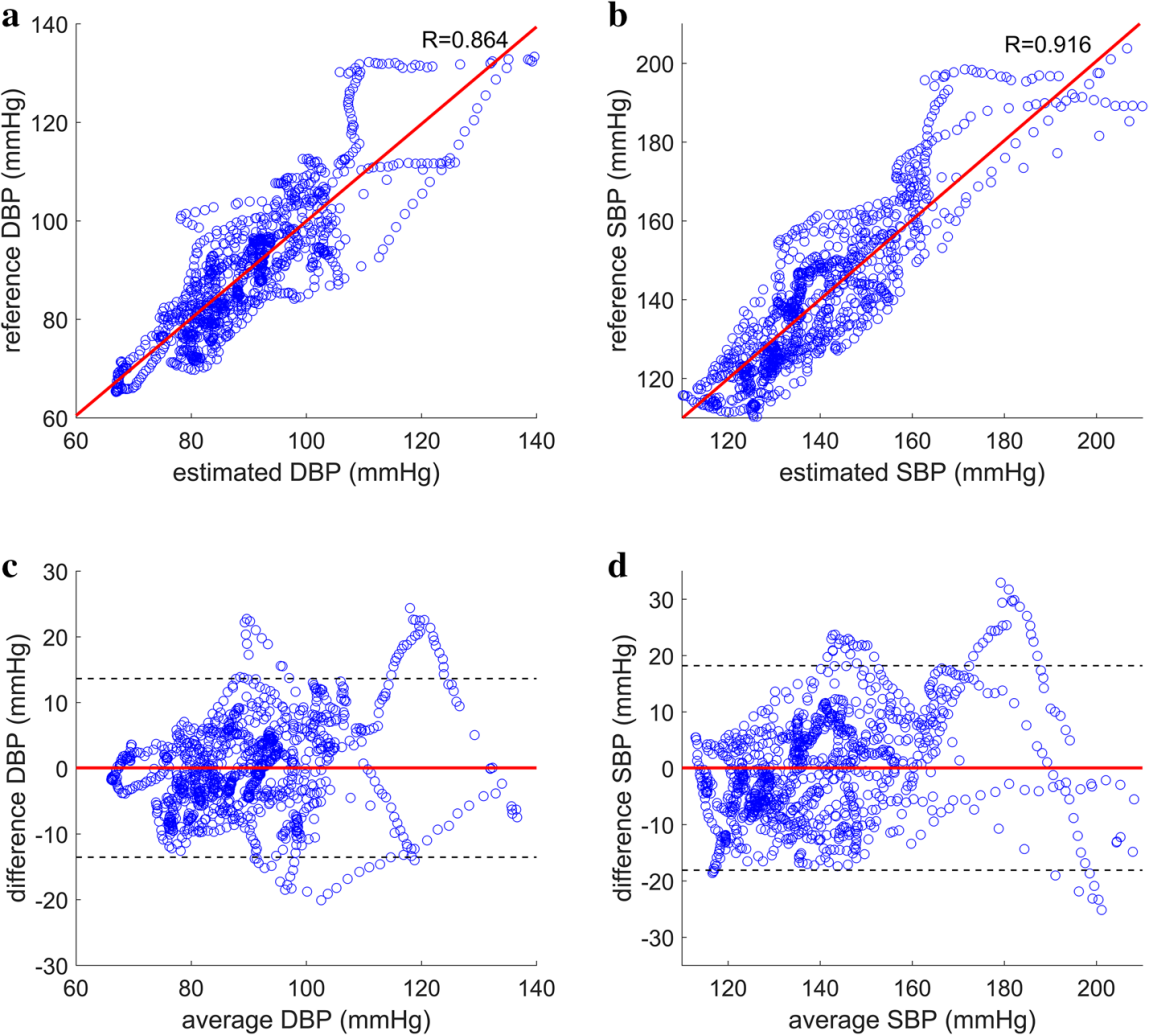
Correlation plots and Bland–Altman plots for DBP and SBP estimation during static squatting exercise. **a** Reference DBP plotted against estimated DBP using the proposed method (R = 0.854). **b** Reference SBP plotted against estimated SBP using the proposed method (R = 0.915). **c** Bland–Altman plot of the estimated DBP using the proposed method with mean difference marked in solid red line and ± 1.96 standard deviation marked in dashed black lines. **d** Bland–Altman plot of the estimated SBP using the proposed method with mean difference marked in solid red line and ± 1.96 standard deviation marked in dashed black lines

**Fig. 6:**
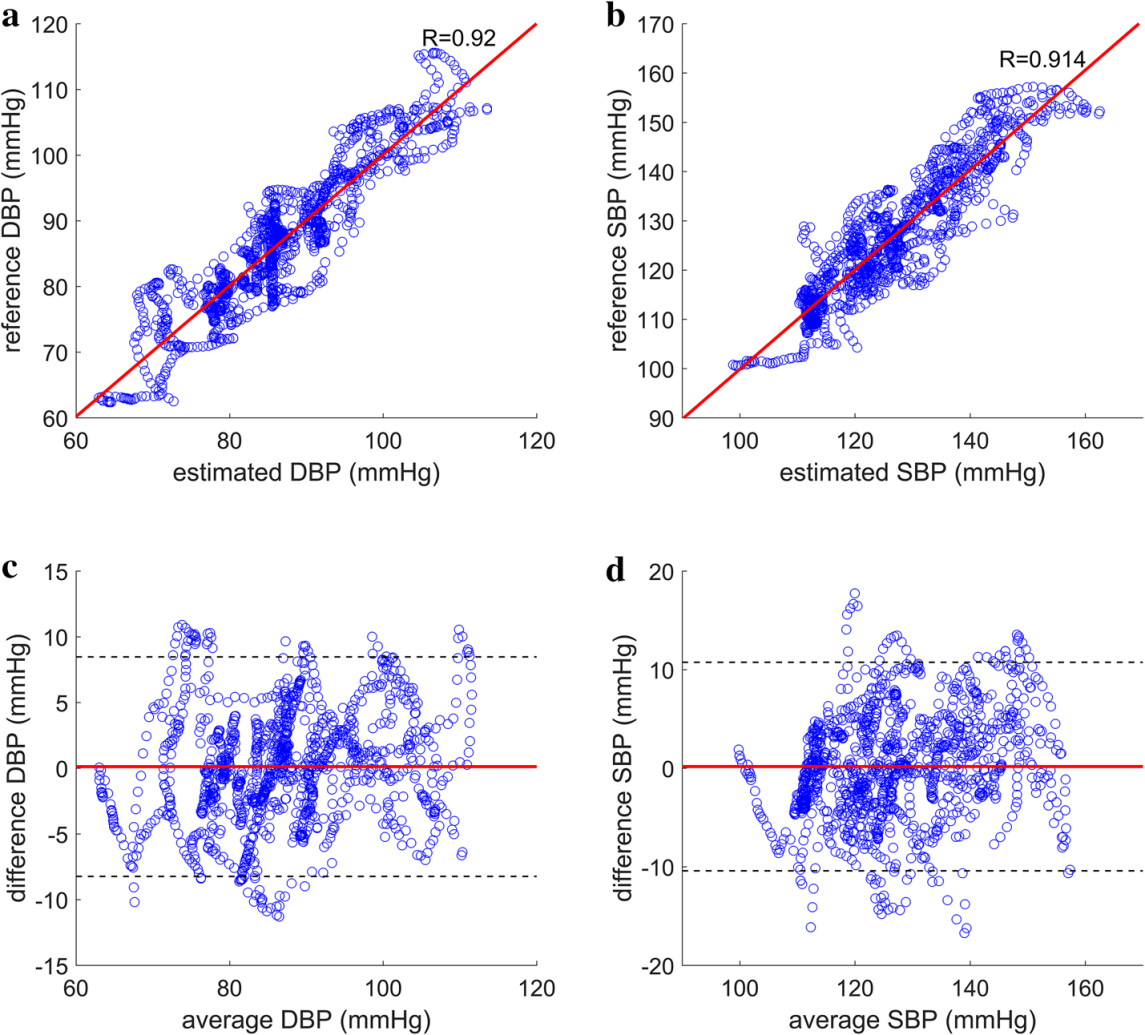
Correlation plots and Bland–Altman plots for DBP and SBP estimation during cold pressor test. **a** Reference DBP plotted against estimated DBP using the proposed method (R = 0.921). **b** Reference SBP plotted against estimated SBP using the proposed method (R = 0.914). **c** Bland–Altman plot of the estimated DBP using the proposed method with mean difference marked in solid red line and ± 1.96 standard deviation marked in dashed black lines. **d** Bland–Altman plot of the estimated SBP using the proposed method with mean difference marked in solid red line and ± 1.96 standard deviation marked in dashed black lines

The proposed method for DBP estimation yielded Pearson’s correlation coefficients of 0.854 and 0.921 for static exercise and cold pressor test, respectively (Figs. 5 and 6) with mean RMSE of 6.31 and 3.87 mmHg across all subjects during static exercise and cold pressor test, respectively. When the estimated DBP was added to the PP estimated using the proposed method to generate estimates of SBP, the correlation coefficients to the reference SBP were 0.915 and 0.914 for static exercise and cold pressor test, respectively (Figs. 5 and 6), and the mean RMSE for SBP was 8.41 and 4.88 mmHg for static exercise and cold pressor test, respectively (Table 2).

**Table 2:** Accuracy of DBP and SBP estimated using the proposed method

Subject	Static exercise RMSE (mmHg)		Cold pressor test RMSE (mmHg)	
	DBP	SBP	DBP	SBP
1	8.55	9.00	4.43	4.81
2	6.18	7.82	6.13	8.22
3	3.04	6.29	3.63	4.40
4	8.10	13.67	5.32	6.80
5	8.65	9.48	1.90	2.44
6	13.90	15.26	6.56	8.60
7	4.54	4.83	3.45	3.18
8	3.41	4.74	3.06	4.32
9	4.53	9.05	1.92	3.84
10	2.44	6.43	4.19	3.76
11	7.10	7.19	2.77	4.40
Mean	6.40 ± 3.35	8.52 ± 3.36	3.94 ± 1.57	4.98 ± 2.01

## Discussion

### Novel method for PP estimation

The proposed method yielded high accuracy for PP estimation in all subjects for both types of hemodynamic interventions. The method considers changes in both SV and C as represented in (2). Given the success of the proposed method, these are likely reflected on the changes in PEP and PTT, as shown in Fig. 2. Throughout the measurement duration, changes in PP are always accompanied by changes in PEP and/or PTT. PEP changes immediately at the end of the exercise phase in this subject, but PTT and PP are delayed and remain relatively stationary until some point after the start of the recovery phase. Furthermore, PTT recovers in a slower manner than PP, overestimating PP when 1/PTT^2^ is the sole parameter. The addition of PEP as a parameter improves the model by reflecting the fast recovery of PEP, bringing the estimated value closer to the reference PP value. The proposed method combining both these parameters improves estimation by incorporating information from both inputs and yielding smaller RMSE.

Efforts to estimate PP in previous studies [20, 21] also yielded high accuracy, but statistically significant improvement on the mean RMSE indicates the addition of information about SV through the integration of PEP has substantially contributed to refining PP prediction. The two previous studies used the Moens–Korteweg and Bramwell–Hill equations derived from the pulse wave propagation model with the assumption that volume change is constant during PTT measurement. This assumption may have been valid for these studies as only a moderate SV change was induced in one study using pedaling [21]. The other study did not induce BP change based on increased SV [20]. However, as previously mentioned, significant SV change usually accompanies significant BP change, and therefore needs to be reflected in PP estimation.

### Changes in estimation accuracy based on intervention type

Static squatting exercise was performed to induce BP change through increased CO, or the product of SV and heart rate (HR), and increased TPR. In such situations, both PEP and PTT should decrease to accommodate the physiological response to the hemodynamic changes. Therefore, the proposed method, which uses linear combination of the two parameters, still yielded a significantly improved estimation with lower RMSE at *p *< 0.01, higher correlation, and narrower limits of agreement compared with the method using 1/PTT^2^ as the sole parameter.

In general, the cold pressor test increases TPR, but the changes in CO are not predictable and may be subject-dependent [41, 42]. In some subjects, PEP decreased with increased PP, showing a similar response pattern as observed in static exercise. In other subjects, PEP fluctuated in a small range over the entire measurement duration without a noticeable pattern associated with changes in PP. In these cases, PP estimation using just 1/PTT^2^ as the sole input parameter may be as accurate as the proposed method. The correlation coefficient for the PTT-based method is much higher for the cold pressor test than the static exercise, indicating better accuracy in this case. However, the proposed method had statistically significant reduction in the mean RMSE at *p *< 0.05, higher correlation coefficient, and narrower limits of agreement for the cold pressor test compared with the PTT-based method. These findings support the importance of adding information about SV even in cases when changes in SV are expected to be small.

### Variability of model coefficients and practicality of the proposed method

Although the purpose of this study was to demonstrate that stroke volume must be incorporated in the estimation of pulse pressure, the individualized models calculated here may lack real-world applicability. Therefore, in order to analyze the variability of the model coefficients derived across each subject and to produce group models applicable to all subjects, we have calculated the mean and the standard deviation of each of the coefficients in our proposed model of PP and the PTT-based model of PP (Table 3). The coefficients *β*_1_ and *β*_2_ have a larger ratio of mean and standard deviation than *m*_1_ for static exercise, indicating that the model coefficients are more variable across subjects for the proposed model of PP. However, when the mean model coefficients were used to calculate the RMSE and the correlation across the subjects as was done previously, the mean RMSE value was similar for both models without statistically significant difference (tested using Student’s *t*-test), while the correlation coefficient was much higher for the proposed model (RMSE 10.5 and 9.5 mmHg, correlation 0.55 and 0.40 for the proposed model and the PTT-based model, respectively). The results are not as clear for the cold pressor test, as there are two outliers for both models. Subjects 6 and 9 have reversed signs for the coefficients of the proposed model, while subjects 1 and 9 have reversed signs for the coefficients of the PTT-based model. These values make the mean and the standard deviation of the coefficients rather unreliable in terms of variability analysis, but when the same RMSE and correlation analysis was done using the mean model coefficients, the proposed model turned out to be superior for cold pressor test as well (RMSE 7.5 and 9.8 mmHg, correlation 0.32 and − 0.21 for the proposed model and the PTT-based model, respectively).

**Table 3:** Model coefficients for the proposed PP model and the PTT-based PP model

Subject	Static exercise					Cold pressor test				
	Proposed method			PTT-based method		Proposed method			PTT-based method	
	*β*_0_	*β*_1_	*β*_2_	*m*_0_	*m*_1_	*β*_0_	*β*_1_	*β*_2_	*m*_0_	*m*_1_
1	45	− 15.2	0.78	32	0.24	38	− 3.3	0.20	52	− 0.10
2	43	− 2.2	0.16	36	0.10	79	− 19.0	0.63	6	0.30
3	38	− 4.9	0.20	27	0.11	43	− 7.6	0.26	33	0.10
4	51	− 6.7	0.33	36	0.19	− 1	− 3.8	0.81	− 5	0.67
5	33	− 4.5	0.24	0	0.24	34	− 1.8	0.09	28	0.05
6	43	− 3.1	0.19	35	0.10	17	5.9	− 0.02	25	0.20
7	39	− 6.7	0.45	31	0.09	47	− 4.8	0.22	23	0.19
8	17	− 3.5	0.31	10	0.22	41	− 0.6	0.10	41	0.07
9	27	− 1.3	0.13	22	0.09	30	27.2	− 1.25	77	− 0.75
10	26	− 0.5	0.05	27	0.02	9	− 1.3	0.23	8	0.13
11	31	− 7.8	0.60	18	0.22	35	− 7.4	0.60	30	0.02
Mean	35.7 ± 10.0	− 5.1 ± 4.1	0.3 ± 0.2	24.9 ± 11.6	0.15 ± 0.07	33.8 ± 21.2	− 1.5 ± 11.3	0.17 ± 0.54	28.9 ± 22.7	0.08 ± 0.34

The results obtained using the mean model coefficients are rather unimpressive, but indicate that the proposed PP model performs better than the PTT-based PP model in practical terms even with larger variations in the model coefficients across the subjects. Although individualized model coefficients yield the desired accuracy, these are limited in that the models must be optimized for each subject, which may not be practical or possible. Many studies have used individualized models, but group-models, such as the one derived here by averaging the model coefficients, with subject-specific calibrations are more practical in terms of real-world applicability. When we use a single-point calibration on our analysis above by shifting the offsets for each model (*β*_0_ and *m*_0_) by the difference in the model output and the first PP measurement, for static exercise we find that the mean RMSE for the proposed PP model reduces to 6.7 mmHg, while the PTT-based model has a mean RMSE of 9.2 mmHg, which yields statistically significant difference using Student’s *t*-test. For cold pressor test using the same calibration method, the mean RMSE across all subjects reduces to 5.5 mmHg for the proposed model and 6.2 mmHg for the PTT-based model. These results are indicative of the performance of the two models in real-world applications. Therefore, though the model coefficients may be more variable for the proposed model of PP as compared to the PTT-based model of PP, when applied in practical terms, the proposed model still performs better especially when changes in stroke volume are involved.

### Improving stroke volume estimation

Although the proposed method yielded a high accuracy for PP estimation in both types of interventions, the use of PEP to approximate SV is a concern as many factors affect SV. Some studies reported a linear relationship between PEP and SV; however, many other studies found a more complex relationship between these parameters. A slightly more complex method would involve the integration of LVET [34], and this is possible using SCG as well. A study reported that the time delay between SCG AO peak and AC peak consistently corresponds to LVET as measured using an echocardiogram [43]; thus, both PEP and LVET can be extracted from the SCG. However, due to unknown reasons, possibly due to the low sensitivity of the accelerometer or the low resolution of the ADC, we could not extract the AC peaks in SCG signals of some subjects. Additionally, other studies proposed using the amplitude information from the SCG measurements as parameters in SV estimation [35]. This idea was not pursued due to the variation in the amplitude based on the positioning of the accelerometer and the subject-to-subject variability in SCG morphology. Hence, there are ways to further improve SV estimation using accelerometer-based SCG, which will result in a more accurate PP estimation. Nonetheless, the focus of this study was to prove that PP estimation can be enhanced using SV approximation, and the results indicate that even a modest incorporation of SV using PEP causes significant improvement.

### Blood pressure estimation accuracy of the proposed method

In terms of standard measurements of BP, DBP, and SBP, the proposed method has achieved high correlations to the reference device. Correlation coefficients for DBP were 0.854 and 0.921 in static exercise and cold pressor test, respectively, whereas the values for SBP were 0.915 and 0.914 in static exercise and cold pressor test, respectively. These values are comparable to those obtained by Ding et al. [20] who achieved 0.88 for DBP and 0.91 for SBP, and to those obtained by Tang et al. [21] who achieved 0.83 for DBP and 0.89 for SBP. With regard to the limits of agreement, Ding et al. achieved SD of 4.06 mmHg for DBP and 5.21 mmHg for SBP, and Tang et al. achieved SD of 5.7 mmHg for DBP and 5.8 mmHg for SBP. The present study achieved a SD of 7.15 mmHg for DBP and 9.30 mmHg for SBP during static exercise and 4.25 mmHg for DBP and 5.39 mmHg for SBP during cold pressor test. Although the SD values are higher for the proposed method in static exercise compared with the values obtained by either of the previous studies, when the range of BP change is considered, the limits of agreement here are reasonable and may even be an improvement upon the two previous studies. Of the two studies, Tang et al. induced greater BP change through pedaling, but the absolute range of BP is only comparable to the results of the cold pressor test in the present study. In the current study, the number of BP recordings and the spread of the BP values are larger. Therefore, the improvement upon the PP estimation by the proposed method and high correlation coefficients for estimated DBP and SBP against the reference values at wide ranges indicate that the proposed method also provides highly accurate DBP and SBP estimation along with highly accurate PP estimation.

### PP and cardiovascular risk

Non-invasive continuous BP monitoring has received considerable attention in the past few decades due to the development of wearable biosensor systems, and the potential for hypertension prevention. The focus has been on DBP and SBP, as the clinical assessment of hypertension depends on these values rather than PP. However, the value of PP in the clinical assessment of cardiovascular risk has become increasingly evident [44], and various studies have verified PP as an independent predictor of cardiovascular risk. In the 2003 Framingham Heart Study on predictors of congestive heart failure, 16 mmHg, or 1 SD from the mean, increase in PP was associated with 55% increased risk [45]. In the 2008 study on the prediction capability of cardiovascular event, central PP was the most powerful independent predictor of myocardial infarction or stroke, whereas mean central BP and peripheral BP were not independently related to the primary endpoint [46]. One study also found that the increase in either central or peripheral PP significantly predicts all-cause and cardiovascular mortality rates [47]. Furthermore, in a study involving 50 hypertensive patients, increase in PP and decrease in the ratio of SV and PP was related to the increase in the crude risk of total cardiovascular events over 10 years [48]. Other studies reported increased PP in normotensive males as an independent determinant of coronary heart disease risk [49]. PP was also reported as a better predictor of cardiovascular events than SBP in patients with type 2 diabetes [50].

In the same period, a growing number of studies have reported on the relevance of BP variability (BPV) for detecting hypertension and cardiovascular risk. In 2014, a study found positive correlation between increased BPV and carotid intima-media thickness [51], and increased intima-media thickness has been known to be associated with increased myocardial infarction and stroke [52]. Additionally, the movement of the carotid artery wall and various BPV parameters have been found to be affected by hypertension risk [53], while PP variability and SBP variability were found to be associated with alterations of large arteries in hypertensive patients [54]. More relevant to the current study, in an analysis of BPV during cold pressure test, subjects with predisposition to hypertension were found to have increased BPV [55]. Finally, a highly regarded review in 2010 discussed the inadequacies of BP based diagnoses and therapies of hypertension, highlighting the clinical relevance of BPV in the prediction of vascular events and in the use of antihypertensive drugs [56]. With regards to PP as a proxy of BPV, these studies further emphasize the growing recognition of PP as a key indicator of cardiovascular risk. As continuous SBP and DBP monitoring has been recommended over single intermittent measurements, the importance of continuous PP measurement has been suggested as well [57]. Therefore, continuous tracking of PP may provide insights into cardiovascular risk independent of abnormal levels of SBP and DBP. The high level of accuracy achieved in the present study compared with SBP or DBP suggests that PP may be a better target for continuous monitoring in terms of cardiovascular risk management.

### Limitations and future work

This study has a few technical limitations. First, as previously mentioned, the estimation of SV using PEP is not an exact method. However, it seems to be successful and improves upon previously available methods for PP estimation. An interesting follow-up study would involve the direct measurement of SV for PP estimation to fully validate the overall method proposed here using (2) and (5), without the use of SV approximation in (3). Second, with regard to PP estimation, central aortic compliance has been equated with the compliance of the arteries traveling from the aorta to the skin of the torso, which consists of both central and peripheral arteries. In terms of experimentation, the use of the Finometer here may have caused errors due to the measurement of peripheral BP values rather than central BP values. The latter BP values are needed for (2), as the ratio of SV and systemic compliance C is approximating central PP rather than peripheral PP. Finally, the study has a relatively small sample size. A larger study with greater anthropomorphic variation is warranted to fully verify the proposed PP estimation method in older subjects and in subjects with abnormal blood pressure levels and increased cardiovascular risk.

## Conclusions

In this study, we proposed a novel method for BP and PP estimation based on PTT and SV approximation using PEP measured continuously and non-invasively. The performance of the proposed method was compared against previous methods. The previous methods used a similar PP estimation method based on arterial compliance but with the assumption of constant volume change. Two types of hemodynamic interventions were used to induce different changes in TPR and SV, and even in the case when SV was expected to stay relatively stable, the proposed method significantly improved upon PP estimation compared with the alternative method. The improved accuracy of PP and SBP estimation indicates that the proposed method in this study may permit better ubiquitous BP monitoring and management of hypertension and other indicators of cardiovascular risk.

## Abbreviations

BP: blood pressure
PTT: pulse transit time
SBP: systolic blood pressure
DBP: diastolic blood pressure
MBP: mean blood pressure
PP: pulse pressure
SV: stroke volume
PEP: pre-ejection period
ECG: electrocardiogram
SCG: seismocardiogram
PPG: photoplethysmogram
C: arterial compliance
TPR: total peripheral resistance
BCG: ballistocardiogram
LVET: left ventricular ejection time
AFE: analog-front-end
SNR: signal-to-noise ratio
LED: light-emitting diodes
PD: photodiodes
AO: aortic opening
ADC: analog-to-digital converter
CO: cardiac output
RMSE: root-mean-square error
SD: standard deviation
HR: heart rate
